# Crystallisation of Wild-Type and Variant Forms of a Recombinant Plant Enzyme β-*d*-Glucan Glucohydrolase from Barley (*Hordeum vulgare* L.) and Preliminary X-ray Analysis

**DOI:** 10.3390/ijms11072759

**Published:** 2010-07-19

**Authors:** Sukanya Luang, James R. Ketudat Cairns, Victor A. Streltsov, Maria Hrmova

**Affiliations:** 1 School of Biochemistry, Institute of Science, Suranaree University of Technology, Nakhon Ratchasima 30000, Thailand; E-Mail: jeab_bc@hotmail.com (S.L.); cairns@sut.ac.th (J.R.K.C.); 2 Molecular and Health Technologies, CSIRO-Commonwealth Scientific Research Organization, Victoria 3052, Australia; E-Mail: victor.streltsov@csiro.au; 3 Australian Centre for Plant Functional Genomics, University of Adelaide, Waite Campus, Glen Osmond, South Australia 5064, Australia

**Keywords:** macro- and cross-seeding, wild-type and mutant protein, X-ray diffraction

## Abstract

Wild-type and variant crystals of a recombinant enzyme β-*d*-glucan glucohydrolase from barley (*Hordeum vulgare* L.) were obtained by macroseeding and cross-seeding with microcrystals obtained from native plant protein. Crystals grew to dimensions of up to 500 × 250 × 375 μm at 277 K in the hanging-drops by vapour-diffusion. Further, the conditions are described that yielded the wild-type crystals with dimensions of 80 × 40 × 60 μm by self-nucleation vapour-diffusion in sitting-drops at 281 K. The wild-type and recombinant crystals prepared by seeding techniques achived full size within 5–14 days, while the wild-type crystals grown by self-nucleation appeared after 30 days and reached their maximum size after another two months. Both the wild-type and recombinant variant crystals, the latter altered in the key catalytic and substrate-binding residues Glu220, Trp434 and Arg158/Glu161 belonged to the *P*4_3_2_1_2 tetragonal space group, *i.e.*, the space group of the native microcrystals was retained in the newly grown recombinant crystals. The crystals diffracted beyond 1.57–1.95 Å and the cell dimensions were between a = b = 99.2–100.8 Å and c = 183.2–183.6 Å. With one molecule in the asymmetric unit, the calculated Matthews coefficients were between 3.4–3.5 Å^3^·Da^−1^ and the solvent contents varied between 63.4% and 64.5%. The macroseeding and cross-seeding techniques are advantageous, where a limited amount of variant proteins precludes screening of crystallisation conditions, or where variant proteins could not be crystallized.

## 1. Introduction

Plant β-*d*-glucan glucohydrolase enzymes are classified in the GH3 family of glycoside hydrolases (http://www.cazy.org/) that currently includes nearly 3,000 entries [1]. The majority of these entries represent nucleotide sequences obtained from sequencing of bacterial genomes. Almost all GH3 entries have two, three or more individually folded domains, while the spatial arrangement of the domains varies [2,3]. A large proportion of the GH3 entries are enzymes that are variously annotated as β-*d*-glucosidases, glucan-1,4-β-*d*-glucosidases, (1,3)-β-*d*-glucan exohydrolases, (1,3;1,4)-β-*d*-glucan exohydrolases, exo-1,3-1,4-β-*d*-glucanases, *N*-acetyl-β-*d*-glucosaminidases, xylan-1,4-β-*d*-xylosidases and α-l-arabinofuranosidases [1]. Although the GH3 family contains predominantly enzymes, their substrate specificities have not been satisfactorily defined and the most of annotations are based on sequence similarities with enzymes for which biochemical data are available [4].

We have focused our attention in the past upon the biochemical and structural characterization of β-*d*-glucan glucohydrolases from higher plants, in particular on barley β-*d*-glucan glucohydrolase, isoenzyme ExoI (designated as HvExoI). This enzyme was crystallized from a native plant source obtained from barley seedlings [5], although the procedure for isolation of a crystallisable quality of HvExoI is both time consuming and technically challenging [6]. Over the past ten years several structures (Protein Data Bank references 1EX1, 1IEQ, 1IEV, 1IEW, 1IEX, 1J8V, 1LQ2, 1X38, 1X39) have been solved from the native HvExoI crystals with substrate analogues and mechanism-based inhibitors to explain the enzyme’s catalytic mechanism and substrate specificity [2,7–10]. However, a few facets of catalytic and substrate binding mechanisms remain to be explained. To this end we are interested in the specific roles of amino acid residues in the vicinity of the catalytic pair and how specific structural determinants at the entrance of the catalytic pocket control spatial dispositions of isomeric oligosaccharides entering the catalytic site. Further, we have been intrigued by so-called substrate-product trafficking events at the mouth of the catalytic site [2,7]. Here, the glucose product of the hydrolytic reaction, which is released from the non-reducing termini of substrates, remains bound to the enzyme’s active site. However, when a new substrate molecule approaches the enzyme, the glucose product diffuses away from the pocket and the incoming substrate enters the active site [4]. Both events are linked and a precise mechanism, how they proceed, is not known. We presume that the incoming substrate binds in the vicinity of the active site and could, by some delicate mechanism, instigate a product for new substrate interchange. We believe that this substrate/product interchange event represents an ideal model system to study, how products and incoming substrates interact in and/or near enzymes’ catalytic sites in general.

Recently, we have reported the high level expression of recombinant HvExoI (designated as rHvExoI) from a codon-optimized HvExoI cDNA in protease-deficient *Pichia pastoris* under low temperature conditions [11]. We expect that rHvExoI could be used as a suitable enzyme model to study the roles of amino acid residues in catalysis and substrate specificity of this enzyme. To this end, we prepared variants of wild-type rHvExoI, altered (into Ala) in the key catalytic and substrate binding residues Glu220, Trp434 and Arg158/Glu161 by site-directed mutagenesis [12]. From the available structural data [2,7–10] we would expect that Glu220, Trp434 and Arg158 are surface exposed, while Glu161 is buried.

In the current work, we describe the techniques and conditions for preparation of the wild-type and variant rHvExoI crystals that showed excellent diffraction parameters. These crystals were prepared by the macroseeding and cross-seeding techniques with microcrystals obtained from a native plant protein. Seeding techniques have previously been used successfully in bio-macromolecular crystallography [13]. The advantage of these techniques is that they use crystals that provide a preformed, regular surface, onto which new molecules may be added in a regular fashion, generally at a lower degree of supersaturation than is required for nucelation [13].

## 2. Results and Discussion

### 2.1. Protein Expression and Purification

**Figure 1.**
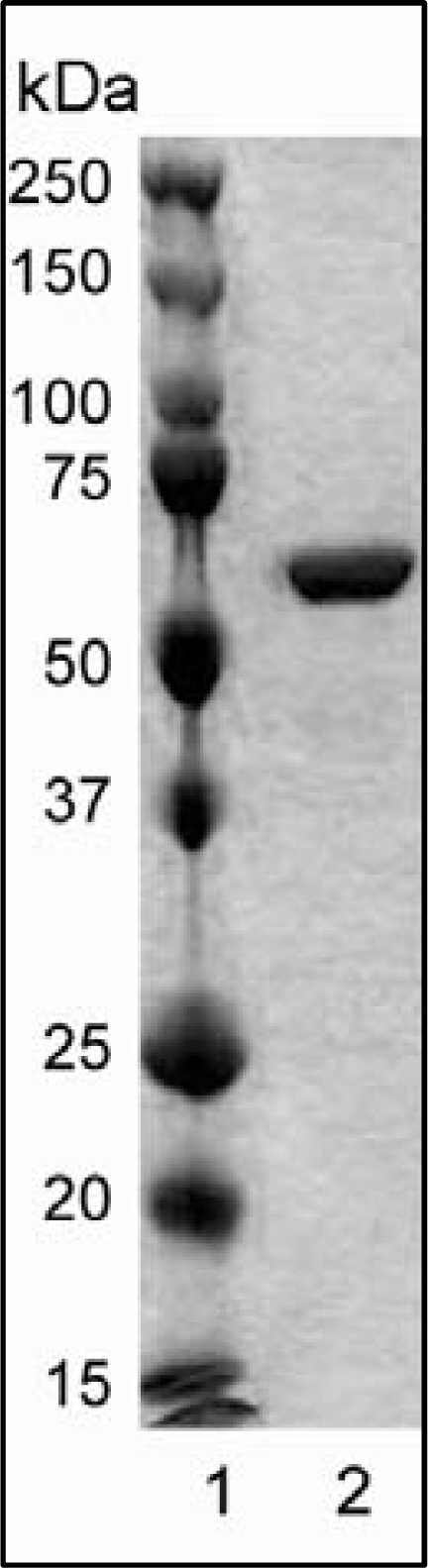
SDS-PAGE profile of recombinant wild-type rHvExoI (lane 2) used for crystallisation by self-nucleation and macro- and cross-seeding using hanging-drop and sitting-drop vapour-diffusion methods. The rHvExoI protein (25 μg) was visualised with a Coomassie Brilliant Blue stain. Protein standards (5 μL of the Precision Plus Protein Standards’ stock) are indicated in lane 1.

Protein expression in *P. pastoris* at a temperature of 293 K, deglycosylation and purification yielded near-homogenous recombinant rHvExoI (Figure 1) with a molecular mass of 67,169 Da [11]. It is of note that rHvExoI contained at its NH_2_-terminus an 8x-His-tag and additional three Ala residues flanking the 8x-His-tag (AHHHHHHHHAA). The Ala residues resulted from the ligation-based cloning process, while the 8x-His-tag was added for protein purification purposes [11]. This 11-residue tag was not removed from the rHvExoI protein that was subjected to crystallisation trials. Expressed rHvExoI was catalytically competent with the catalytic efficiency value k_cat_·K_M_^−1^ = 14 mM^−1^·s^−1^ towards 4-nitrophenyl β-*d*-glucoside. This second-order rate constant was similar to that reported for native fully N-glycosylated HvExoI [14] and a recombinant N-deglycosylated form [11]. Further, the biophysical properties of rHvExoI such as pH optimum and thermostability were also similar to those reported for native or recombinant HvExoI [11,14].

### 2.2. Protein Crystallisation

**Figure 2.**
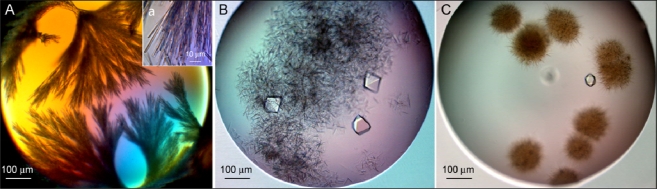
Crystals of recombinant wild-type rHvExoI grown by self-nucleation. Various forms of crystals grown for 14 (**A**) and 97 (**B**–**C**) days using a microbatch-under-paraffin-oil technique from 1.6 M magnesium sulfate, pH 6.5 at 277 K (A) or obtained using sitting-drop vapour-diffusion from 1.8 (B) or 2.2 M (C) ammonium sulfate, both at pH 5 and 281 K. The crystals in (A) formed thin needles of approximately 40–50 μm in their longest dimensions (inset), while in B and C short thin needles are shown that appeared within 14 days and after another 30 days the truncated bi-pyramidal crystals grew that reached dimensions of 80 × 40 × 60 μm after 97 days.

**Figure 3.**
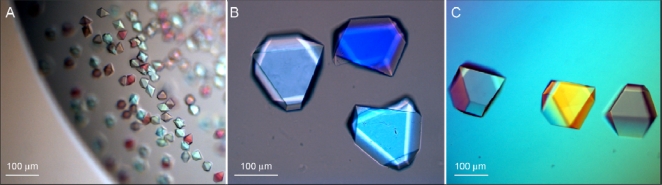
Microcrystals (10 × 5 × 7.5 μm to 20 × 10 × 15 μm) of native HvExoI (**A**) used to grow the recombinant wild-type (**B**) and variant Glu220Ala (**C**) rHvExoI crystals that grew to their full-sizes within 5–14 days. The crystals obtained by seeding in hanging-drops by vapour-diffusion grew to dimensions of up to 500 × 250 × 375 μm at 277 K.

The concentrated rHvExoI was subjected to four types of crystallisation trials (Figures 2 and 3). We firstly set-up the microbatch under paraffin oil trials at 277 K and 287 K with the goal to screen approximately 130 independent conditions, including those that were previously found to be successful with a native plant enzyme [5]. These conditions (1.7 M ammonium sulfate, 75 mM HEPES-NaOH buffer, pH 7, containing 7.5 mM sodium acetate and 1.2% (w/v) PEG 400) previously produced crystals of native plant HvExoI that belonged to the primitive tetragonal space group P4_3_2_1_2 and yielded high-resolution diffraction patterns [2,5,7]. Nevertheless, despite intensive effort, no diffraction-quality crystals grew, and after approximately 14 days only the formation of a highly intricate interlaced web of thin needles of approximately 40–60 μm in their longest dimensions was observed. Figure 2A and its inset show the appearance of these needles that grew from 1.6 M magnesium sulfate at pH 6.5 and 277 K.

Our second approach to growing the diffraction quality crystals led us to set-up close to 400 trials at the Bio21 Collaborative Crystallisation Centre at 281 K and 293 K by a sitting-drop vapour-diffusion method. After approximately 21 days at 281 K, we could observe formation of short thin needle-shaped crystals that either remained dispersed throughout the droplets (Figure 2B) or formed well-organised round balls (Figure 2C). However, after about a month, in some of the droplets with needles (Figures 2B and 2C) truncated bi-pyramidal crystals formed, which reached dimensions of 80 × 40 × 60 μm after about 97 days (Figure 2B). It was of note that these crystals were only observed at 281 K in droplets with 1.6 to 2.2 M ammonium sulfate, containing 10 mM malate-MES-Tris buffer, pH 5. These conditions were similar to the conditions that we found previously for native HvExoI [5], except that here the pH value of 5 was more acidic then that used previously (pH 7), and that the protein concentration was almost twice as high (12.5 mg·mL^−1^ *versus* 6.8 mg·ml^−1^). We expected that these truncated bi-pyramidal crystals could belong to the tetragonal P4_3_2_1_2 space group found for the native protein crystals [5], although at this stage we were not able to collect their diffraction patterns. Notably, in an identical screen at a higher temperature of 293 K the bi-pyramidal crystals were not formed, so it seemed that the temperature was a critical factor for crystal formation of rHvExoI. Thirdly, we also attempted to grow crystals by self-nucleation at 277 K by hanging-drop vapour diffusion and using the conditions developed for native HvExoI [5]. However, we could not observe crystal formation within 180 days and rHvExoI mostly precipitated or the drops remained clear.

As no diffraction-quality crystals were obtained with the three crystallisation approaches described above, we turned our attention to seeding at 277 K using a hanging-drop vapour-diffusion method that was used successfully for preparation of large well-diffracting crystals of native HvExoI [7–10]. We first examined, if we could use microcrystals obtained from native HvExoI to seed the wild-type recombinant rHvExoI protein, despite the differences between the two proteins. These differences included the 11 additional residues of the affinity tag at the NH_2_-terminus, as described above. Further, the rHvExoI protein was N-deglycosylated by endoglycosidase H, such that only one N-linked N-acetyl-β-*d*-glucosaminyl residue [12] remained attached to each of the three N-glycosylation sites Asn221, Asn498 and Asn600 [2,11]. Approximately 48 h after rHvExoI was macroseeded with the native microcrystals of the sizes between 10 × 5 × 7.5 μm and 20 × 10 × 15 μm, the original native microcrystals started growing in size. The fully grown crystals of wild-type rHvExoI reached dimensions that varied between 100 × 50 × 75 μm (Figure 3B) and 500 × 250 × 375 μm after 5 to 7 days and these crystals had a similar bi-pyramidal morphology as the native microcrystals (Figures 3A). Having succeeded in growing wild-type crystals, using native microcrystal seeds, we prepared large recombinant crystals of three rHvExoI variants, specifically Glu220Ala (Figure 3C), Trp434Ala and of the double mutant Arg158Ala/Glu161Ala, also using native seed crystals. The newly grown variant crystals reached sizes of 100 × 50 × 75 μm (Figure 3C) to 500 × 250 × 375 μm and showed a similar bi-pyramidal morphology as the native microcrystals. The variant crystals grew slightly slower and reached their maximum dimensions after 10–14 days.

### 2.3. X-ray Diffraction

**Figure 4.**
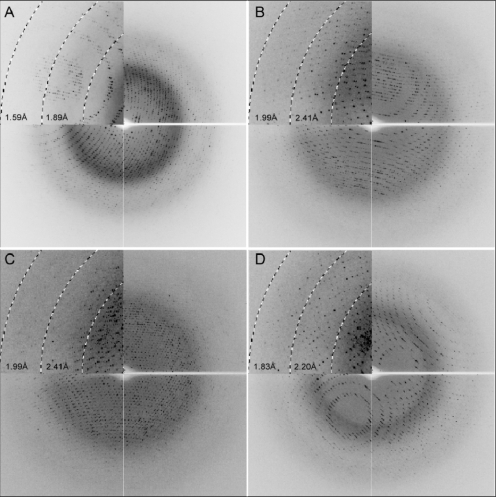
X-ray diffraction patterns of the recombinant wild-type (**A**), and variant Glu220Ala (**B**), Trp434Ala (**C**) and Arg158Ala/Glu161Ala (**D**) rHvExoI crystals. The left top insets show diffraction intensities to 1.59–1.89 Å (A), 1.99–2.41 Å (B and C) and 1.83–2.20 Å (D).

Single wild-type and variant rHvExoI crystals were cryo-protected and subjected to diffraction at the MX1 beamline of the Australian Synchrotron. All of the X-ray diffraction data sets were virtually complete beyond 1.57–1.95 Å (Figure 4; Table 1). The HKL2000 indexing and systematic absences calculated that the space groups of the wild-type and variant rHvExoI crystals were consistent with a primitive tetragonal space group P4_3_2_1_2 (Figure 4 and Table 1), and as expected, these space group characteristics were similar to their native counterparts [2,7]. In line with these observations was our previous finding that the native HvExoI microcrystals of the sizes 20 × 10 × 15 μm diffracted beyond 2.80 Å on an in-house rotating anode X-ray source and belonged to a primitive tetragonal space group P4_3_2_1_2 [15]. Thus, our data are in agreement with other reports, where the space group characteristics of macroseeds and fully grown crystals were identical [16–18], or that as a bonus, the resolution of the newly grown crystals has improved [18]. On the contrary, other authors have reported that during seeding trials mutant crystals often crystallized in different space groups than their macroseeds [19].

The best diffraction data, to 1.57 Å were collected from the wild-type crystals, followed by the Arg158Ala/Glu161Ala, Glu220Ala and Trp434Ala variants that diffracted to 1.65 Å, 1.90 Å and 1.95 Å, respectively (Figure 4, Table 1). The lattice dimensions of wild-type and variant crystals varied between a = b = 99.2–100.8 Å and c = 183.2–183.6 Å, and there appeared to be one molecule in the asymmetric units, according to the Matthews coefficient calculation [20]. The Matthews coefficients of recombinant crystals were between 3.4–3.5 Å^3^·Da^−1^ with solvent contents of 63.4% to 64.5%. The R_merge_ values of 5.6% to 10.1% were obtained with <I/σ/(I)> of 37.0 to 71.2, whereas the multiplicity of the individual datasets was well above 20 and varied between 26 to 29 (Table 1). It was of note that the completeness for the highest resolution shells of the wild-type and Glu220Ala datasets was lower, despite the high multiplicity and crystal symmetry, because the data were integrated into the corners of the detector. Also, the mean <I/σ/(I)> values for these datasets indicated that they actually diffracted to higher resolution than that stated in Table 2. It was not surprising that the most favourable diffraction statistics was obtained with the wild-type crystals that were seeded with the wild-type native macroseeds (Figure 4 and Table 1).

## 3. Experimental Section

### 3.1. Expression and Purification of Wild-Type and Variant Forms of rHvExoI

Wild-type (GenBank accession No. GU441535) and variant codon-optimized cDNAs, encoding a mature barley β-*d*-glucan exohydrolase I (HvExoI) inserted in pPICZαBNH_8_ expression vectors, were expressed in *P. pastoris*, strain SMD1168H and purified by ion exchange, immobilized metal affinity chromatography (IMAC), *N*-deglycosylation by endoglycosidase H and a second round of IMAC, as described previously [11]. The variant forms included Glu220Ala, Trp434Ala and a double mutant Arg158Ala/Glu161Ala, for which the constructions of the cDNA fusions will be described elsewhere [12]. At the final purification step before crystallisation, the *N*-deglycosylated rHvExoI wild-type and variant enzymes were eluted from a BioGel-P100 size-exclusion column with 50 mM sodium acetate buffer, pH 5.25 containing 200 mM NaCl and 1 mM dithiothreitol at a liner flow rate of 0.5 cm·h^−1^. The protein purities of the rHvExoI fractions were analyzed by SDS-PAGE, using 12.5% w/v polyacrylamide and bis-polyacrylamide gels and standard techniques [6]. The protein concentration was estimated with a Bio-Rad protein assay kit (Bio-Rad Laboratories, Gladesville, New South Wales, Australia) using bovine serum albumin (Sigma Chemical Company, St. Louis, MO, USA) as a standard. The protein standards ‘Precision Plus Protein Standards’ used for SDS-PAGE were from Bio-Rad Laboratories.

### 3.2. Enzyme Assays

The activities of pooled and concentrated (using 10 kDa cut-off centrifugal filter units (Millipore, Bedford, MA, USA) wild-type and variant enzymes were assayed against 4-nitrophenyl β-*d*-glucopyranoside (Sigma) in 50 mM sodium acetate buffer, pH 5.25.

### 3.3. Crystallisation of Wild-Type and Variant Forms of rHvExoI

Near-homogenous N-deglycosylated wild-type and variant rHvExoI proteins were concentrated to 12.5 mg·mL^−1^ in 20 mM sodium acetate pH 5.25 and filtered through a 0.22 μm filter (Millipore). Screening of crystallisation conditions was performed by four experimental approaches. Firstly, initial crystallisation conditions were screened using a microbatch under paraffin oil technique. Here, 1 μL of precipitant solutions and 1 μL of the solution containing 12.5 mg·mL^−1^ of rHvExoI were added into 10 μL of 100% Paraffin oil (Hampton Research, Aliso Viejo, CA, USA) that was previously dispensed in microbatch 72 well Greiner (Terasaki style) plates (Hampton Research). The formulations of Crystal Screen 2, Crystal Screen Lite and Grid Screen Ammonium Sulfate (Hampton Research) were used as precipitants and the crystals grew at 277 K or 287 K in a vibration-free crystallographic cabinet (Molecular Dimensions, Suffolk, UK). Secondly, crystallisation trials were set-up robotically (Phoenix Nano-Dispenser, Art Robbins Instruments, Sunnyvale, CA, USA) in sitting drops, in which 300 nL droplets of rHvExoI were mixed with the same volumes of precipitants and crystal growth proceeded at 281 K and 293 K at the Bio21 Collaborative Crystallisation Centre (CSIRO, Parkville, Australia) [21]. The precipitants from the PSS_1_Com5 and PS gradient-mid range formulation screens were used, whereas both screens were prepared in-house at the Bio21 Centre (http://www.csiro.au/c3/Facility/c3_centre_robotic_crystal.htm), following the recommendation from Emerald BioSystems (Bainbridge Island, WA, USA) for the first screen and those reported by Newman [22] for the second screen. The PSS_1_Com5 screen uses the inorganic precipitants such as sulfates, chlorides, citrates and phosphates, but also 2-methyl-2,4-pentanediol, glycerol and polyethyleneglycols (PEGs) in the pH ranges of 5–8.7. On the other hand, the PS gradient-mid range formulation relies on ammonium sulfate and sodium malonate as precipitants in a 10 mM malate-MES-Tris buffer system in the pH ranges of 4.5–9 [22]. Thirdly, attempts were made to grow crystals under the conditions developed for native HvExoI at 277 K using a hanging-drop vapour diffusion method [5]. Lastly, and most importantly, the rHvExoI crystals were grown in hanging drops at 277 K that were seeded with the native HvExoI microcrystals prepared as described previously [5]. The sizes of the microcrystals for the latter conditions varied between 10 × 5 × 7.5 μm and 20 × 10 × 15 μm. The hanging drops were prepared at 277 K as follows. The volume of 4 to 6 μL of rHvExoI at 12.5 mg·mL^−1^ was added to 4 μL of the precipitant solution A (100 mM HEPES-NaOH buffer pH 7, 2.4% (w/v) PEG 400, 1.6 M ammonium sulfate) on 22-mm siliconized circular glass cover slips (Hampton Research). A few microcrystals of native HvExoI were transferred into the hanging drop with a cat whisker. Here, the whisker gently touched the surface of a macroseed stock of native HvExoI and subsequently the whisker was swiftly immersed into a new rHvExoI drop. The cover slips with the seeded hanging drops were placed over 1 mL of reservoir solutions (1.7 M ammonium sulfate in 50 mM HEPES-NaOH buffer, pH 7) contained in the 24 well Linbro plates (Hampton Research), and the wells were sealed with vacuum grease (Dow Corning Corporation, Midland, MI, USA). Crystals from the seeded drops appeared within 5–14 days and were suitable for X-ray data collection. The crystals were photographed through a Leica Laser Microdissection microscope (Leica, North Ryde, Australia) equipped with fluorescence and differential interference contrast.

### 3.4. X-ray Data Collection and Processing

Single enzyme crystals with the longest dimensions of 100 to 500 μm were cryo-protected in 20% (v/v) glycerol concentration in solution A (as specified above in Section 3.3.) [10] and flash cooled in the cold N_2_ stream at the beamline MX1 of the Australian Synchrotron. X-ray diffraction data sets were collected at 0.5° oscillations (1 sec exposures) through 360° on the ADSC Quantum 210r Detector [23]. The data were processed with the HKL2000 suite of programs [24].

## 4. Conclusions

In summary, excellent X-ray diffraction data were obtained from the recombinant wild-type and variant rHvExoI crystals grown by seeding from a native plant source protein in hanging-drops by vapour-diffusion. The recombinant crystals grew relatively fast and within 5–14 days reached dimensions of up to 500 × 250 × 375 μm. The fully grown recombinant crystals retained the space group characteristics of their native macroseeds and diffracted beyond 1.57 Å to 1.95 Å. As reported for other proteins, this technique could be valuable, where a limited amount of variant proteins is available precluding crystallisation trials, or where variant protein forms could not be crystallized. We project that cross-seeding using native protein as a source of microcrystals could be successfully used for generation of large recombinant wild-type and variant crystals that could potentially yield high resolution diffraction patterns. Lastly, the diffraction data collected from the wild-type and variant rHvExoI crystals reported here could be used successfully for structure solution. The structural data are currently being prepared for publication [12].

## Figures and Tables

**Table 1.** Data collection statistics from the wild-type and variant rHvExoI crystals, calculated by the HKL2000 suite of programs.

	**Wild-type**	**Glu220Ala**	**Trp434Ala**	**Arg158Ala/Glu161Ala**
Unique reflections	119968	74171	64460	107601
Resolution a (Å)	1.57 (1.6−1.57)	1.90 (1.94−1.90)	1.95 (1.98−1.95)	1.65 (1.68−1.65)
Mean multiplicity a,b	29 (26)	27 (16)	27 (16)	26 (12)
Completeness a,b (%)	99 (86)	99 (88)	100 (100)	99.8 (98)
Mean <I/σ/(I)> a	71.2 (5.6)	54.8 (4.3)	37.0 (2.2)	58.7 (2.3)
R_merge_^a^–^c^ (%)	6.7 (47)	8.9 (64)	10.1 (87)	5.6 (82)
a = b (Å) d	99.2	100.2	100.1	100.8
c (Å) d	183.5	183.2	183.6	183.2

a Numbers in parenthesis represent the values in the highest resolution shell.

b The numbers were rounded to no decimal place.

c R_merge_ = 100[∑(I_i_-<I>)^2^/∑I_i_^2^] is summed over all independent reflections.

d The numbers were rounded to the 1^st^ decimal place.
